# The Role of Nutrition in Osteoarthritis Development

**DOI:** 10.3390/nu15204336

**Published:** 2023-10-12

**Authors:** Antonia Sophocleous

**Affiliations:** Department of Life Sciences, School of Sciences, European University Cyprus, Nicosia 2404, Cyprus; a.sophocleous@euc.ac.cy

Osteoarthritis (OA) prevalence has increased 113% since 1990, and currently more than half a billion people worldwide are living with this slowly progressing, degenerative joint disease [1]. This figure is expected to continue to rise globally, as population ages and obesity rates increase. People with OA suffer from joint stiffness and deformity and experience chronic pain that can be debilitating [2]. OA poses a substantial economic burden not only on patients but also on society [3], and it has been labelled as a ‘serious disease’ by the U.S. Food and Drug Administration following the submission of a white paper by the Osteoarthritis Research Society International (OARSI) [4].

OA is thought to have a multifactorial aetiology, with factors such as age, obesity, mechanical loading, inflammation, joint injury, and genetic predisposition playing an important role in its pathogenesis [2]. However, the detailed mechanisms underlying OA initiation and development are incompletely understood, and currently no interventions are available to effectively delay disease progression or to restore degraded cartilage [5]. Current medical management is primarily based on trying to control pain, whereas non-pharmacological practices focus on weight loss and physical activity. For people with advanced OA, a surgical intervention could also be a treatment option [6]. Recently, there has been increasing interest in the potential benefits of diet and nutrition on the prevention and management of OA.

Obesity is one of the most significant modifiable risks of OA, not only because of the altered biomechanics of the joint but also due to the release of key inflammatory factors by adipose tissue, resulting in low-grade chronic inflammation [7]. Studies exploring the effects of a fatty-acid-enriched diet on articular joints revealed a tight correlation between inflammation and an increased omega-6/omega-3 polyunsaturated fatty acids (PUFAs) ratio [8]. The overconsumption of omega-6 PUFAs is highly associated with synovitis and cartilage degradation in obese patients, resulting from chronic inflammation [9]. Conversely, omega-3 PUFAs-enriched diets reduce systemic inflammation [10], relieve pain, and improve joint function in patients with OA [11]. Therefore, modulating dietary supplementation in favour of omega-3 PUFAs is potentially a new preventive and therapeutic strategy for joint preservation in obesity-associated osteoarthritis [7].

Another target for treating the OA of obesity is the gut microbiome. The increased systemic inflammation which drives the impact of obesity on OA is now understood to be caused by shifts in the gut microbiome [12]. Schott and colleagues (2018) showed that obesity-related dysbiosis of the gut microbiome can be treated by restoring a healthy microbial community [13]. By strategically manipulating specific microbial species inhabiting the intestine, with the nondigestible fibre oligofructose as a dietary prebiotic supplement, Schott et al. restored the lean gut microbiome profile in obese mice [13]. These mice showed reduced systemic inflammation and ultimately were protected against cartilage loss, suggesting a novel approach to treat obesity-associated OA [13].

Gut microbiome manipulation also has the potential to exert a disease-modifying effect in OA associated with the destabilisation of medial meniscus (DMM). Using the DMM mouse model, we recently showed that treatment with a cocktail of probiotic strains (*Lacticaseibacillus paracasei* 8700:2, *Lactiplantibacillus plantarum* HEAL9, and *L. plantarum* HEAL19) following faecal microbiota transplantation (FMT) in mice, where the microbiome has been depleted, prevents DMM-induced cartilage damage and has a positive impact on the structure of subchondral bone, particularly at the femoral condyle [14].

Vitamin D, primarily known for being vital for bone, teeth, and muscle health, functions by enhancing intestinal calcium and phosphorus absorption [15]. Vitamin D is metabolised to its active metabolite, 1,25-dihydroxyvitamin D [1,25(OH)2D], by a double hydroxylation in the liver and kidney [15]. To elicit mineral transport stimulation in the intestine, 1,25(OH)2D binds to a Vitamin D receptor (VDR) and together causes the transcription of specific genes that encode calcium and phosphorus transport proteins [15]. Interestingly, in the context of OA, long bone joints of VDR knockout mice lack the OA phenotype [16] despite the fact that rickets and osteomalacia are seen right after the first month of life [16]. VDR is expressed in the articular cartilage of OA patients but not in that of healthy volunteers [17]. This suggests that 1,25(OH)2D may affect OA cartilage directly, without ruling out the possibility of 1,25(OH)2D influencing healthy cartilage indirectly through the endocrine system [18]. Although reviews of the literature do not support the beneficial effect of 1,25(OH)2D on radiologic OA, or cartilage volume loss in subjects with sufficient vitamin status (≥50 nmol/L) [19], randomised controlled trials showed that 1,25(OH)2D supplementation might alleviate pain and possibly radiologic OA in patients with a lower vitamin D status (<50 nmol/L) [20].

Vitamin K is a family of similar, fat-soluble compounds, including phylloquinone (vitamin K1) and a series of menaquinones (vitamin K2), that are naturally present primarily in green leafy vegetables (vitamin K1), and to a lesser extent in various animal-based and fermented foods (vitamin K2) [21]. Menaquinones are also produced by the human intestinal microflora. Vitamin K is a cofactor to γ-glutamyl carboxylase, an enzyme that introduces post-translation modification, forming a γ-carboxyglutamate (Gla) domain on proteins [22]. Such proteins are known as vitamin-K-dependent Gla proteins and are involved in physiological processes, such as blood-clotting and calcification [23]. The matrix Gla protein (MGP) involved in calcification is expressed in chondrocytes and acts as an inhibitor to calcification mediated by bone morphogenetic protein-2 [24]. A MGP polymorphism was shown to be associated with radiographic hand OA [25], and insufficient vitamin K intake was shown to impact chondrocyte differentiation and endochondral bone formation [26]. For these reasons, vitamin K is thought to be a potential agent in preventing OA. Evidence thus far through case–control, cross-sectional, and prospective studies suggests that a sufficient level of vitamin K is associated with a lower risk of OA development and pathological joint features [23]. Although a clinical trial investigating the effects of vitamin K on OA patients showed that vitamin K1 did not improve the occurrence of hand OA or osteophyte formation, it did benefit joint space narrowing in patients with insufficient vitamin K at baseline [27]. Without a doubt, more clinical trials investigating the impact of vitamin K on OA symptoms are warranted.

Antioxidant supplements, such as vitamin C, vitamin E, and curcumin, have also been proposed to reduce OA symptoms. Antioxidants counteract oxidative stress by scavenging and neutralising free radicals that are generated by normal biological processes [28]. Most animal studies involving vitamin C demonstrated a benefit in reducing OA symptoms when administered as a diet supplement or as intra-articular injections, and several prospective and cross-sectional clinical studies illustrated vitamin C’s chondroprotective ability. Other studies, however, showed that excess vitamin C may lead to detrimental effects within the body, indicating that the therapeutic potential of vitamin C in managing OA remains ambiguous (reviewed in [29]). Due to its antioxidant and anti-inflammatory effects, vitamin E has also been explored as a potential agent to prevent or treat OA. Data, however, remain controversial because although most studies report a positive relationship between vitamin E and joint health, others reported a negligible or even a negative relationship (reviewed in [30]). Recent clinical studies showed that curcumin, at 160 and 2000 mg/day, was found to be effective in alleviating knee OA symptoms; it demonstrated comparable efficacy and even better tolerability to nonsteroidal anti-inflammatory agents [31,32].

The Impact of trace elements on OA has been recently reviewed by Li and colleagues [33]. Here, authors concluded that while several trace elements such as boron, selenium, and copper increase cartilage matrix formation, enhance chondrocyte proliferation, and have anti-inflammatory and antioxidant effects, other trace elements such as cadmium and iron may exacerbate OA pathogenesis and progression [33].

Countless studies in the wider scientific literature reporting on the relationship between nutrition and OA have been critical in furthering clinicians’ and scientists’ knowledge alike to help combat the initiation and progression of OA. This Special Issue welcomes studies summarizing the current evidence on nutrition and OA interaction. Strengthening the scientific knowledge regarding the mechanisms by which nutritional supplementation protects against OA is crucial for studying new intervention strategies. Such findings will not only cast new light on nutrition and its relationship with OA pathogenesis, but may also help pave the way for new forms of therapy based on dietary manipulation.

## References

[1] GBD 2019 Diseases and Injuries Collaborators (2020). Global burden of 369 diseases and injuries in 204 countries and territories, 1990–2019: A systematic analysis for the Global Burden of Disease Study 2019. Lancet.

[2] Cicuttini F.M., Spector T.D. (1996). Genetics of osteoarthritis. Ann. Rheum. Dis..

[3] March L., Cross M., Lo C., Arden N.K., Gates L., Leyland K., Hawker G., King L., Leyland K. (2016). Osteoarthritis: A Serious Disease: Submitted to the U.S. Food and Drug Administration.

[4] Arden N., Hawker G. (2018). The OARSI white paper explained. Osteoarthr Cartil..

[5] Chen D., Shen J., Zhao W., Wang T., Han L., Hamilton J.L., Im H.-J. (2017). Osteoarthritis: Toward a comprehensive understanding of pathological mechanism. Bone Res..

[6] Rönn K., Reischl N., Gautier E., Jacobi M. (2011). Current Surgical Treatment of Knee Osteoarthritis. Arthritis.

[7] Gambari L., Cellamare A., Grassi F., Grigolo B., Panciera A., Ruffilli A., Faldini C., Desando G. (2023). Targeting the Inflammatory Hallmarks of Obesity-Associated Osteoarthritis: Towards Nutraceutical-Oriented Preventive and Complementary Therapeutic Strategies Based on n-3 Polyunsaturated Fatty Acids. Int. J. Mol. Sci..

[8] Liput K.P., Lepczyński A., Ogłuszka M., Nawrocka A., Poławska E., Grzesiak A., Ślaska B., Pareek C.S., Czarnik U., Pierzchała M. (2021). Effects of dietary n–3 and n–6 polyunsaturated fatty acids in inflammation and cancerogenesis. Int. J. Mol. Sci..

[9] Patterson E., Wall R., Fitzgerald G.F., Ross R.P., Stanton C. (2012). Health implications of high dietary omega-6 polyunsaturated fatty acids. J. Nutr. Metab..

[10] Gil Á. (2002). Polyunsaturated fatty acids and inflammatory diseases. Biomed. Pharmacother..

[11] Deng W., Yi Z., Yin E., Lu R., You H., Yuan X. (2023). Effect of omega-3 polyunsaturated fatty acids supplementation for patients with osteoarthritis: A meta-analysis. J. Orthop. Surg. Res..

[12] Portune K.J., Benítez-Páez A., Del Pulgar E.M.G., Cerrudo V., Sanz Y. (2017). Gut microbiota, diet, and obesity-related disorders—The good, the bad, and the future challenges. Mol. Nutr. Food Res..

[13] Schott E.M., Farnsworth C.W., Grier A., Lillis J.A., Soniwala S., Dadourian G.H., Bell R.D., Doolittle M.L., Villani D.A., Awad H. (2018). Targeting the gut microbiome to treat the osteoarthritis of obesity. JCI Insight.

[14] Sophocleous A., Azfer A., Huesa C., Stylianou E., Blanco G.R., Ralston S.H. (2020). Probiotics prevent cartilage damage and progression of osteoarthritis in mice. Bone Rep..

[15] DeLuca H.F. (1986). The metabolism and functions of vitamin D. Adv. Exp. Med. Biol..

[16] Li Y.C., Pirro A.E., Amling M., Delling G., Baron R., Bronson R., Demay M.B. (1997). Targeted ablation of the vitamin D receptor: An animal model of vitamin D-dependent rickets type II with alopecia. Proc. Natl. Acad. Sci. USA.

[17] Tetlow L.C., Woolley D.E. (2001). Expression of vitamin D receptors and matrix metalloproteinases in osteoarthritic cartilage and human articular chondrocytes in vitro. Osteoarthr. Cartil..

[18] Park C.Y. (2019). Vitamin D in the prevention and treatment of osteoarthritis: From clinical interventions to cellular evidence. Nutrients.

[19] McAlindon T., LaValley M., Schneider E., Nuite M., Lee J.Y., Price L.L., Lo G., Dawson-Hughes B. (2013). Effect of vitamin D supplementation on progression of knee pain and cartilage volume loss in patients with symptomatic osteoarthritis: A randomized controlled trial. JAMA.

[20] Sanghi D., Mishra A., Sharma A.C., Singh A., Natu S.M., Agarwal S., Srivastava R.N. (2013). Does vitamin D improve osteoarthritis of the knee: A randomized controlled pilot trial. Clin. Orthop. Relat. Res..

[21] National Institutes of Health O of Dietary Supplements (2021). Vitamin K—Fact Sheet for Health Professionals. https://ods.od.nih.gov/factsheets/VitaminK-HealthProfessional/#:~:text=Vitamin%20K%20is%20present%20in,K%20circulate%20in%20the%20blood..

[22] Marriott B.P., Birt D.F., Stallings V.A., Yates A.A. (2020). Present Knowledge in Nutrition: Basic Nutrition and Metabolism.

[23] Chin K.Y. (2020). The relationship between vitamin k and osteoarthritis: A review of current evidence. Nutrients.

[24] Xue W., Wallin R., Olmsted-Davis E.A., Borrás T. (2006). Matrix GLA protein function in human trabecular meshwork cells: Inhibition of BMP2-induced calcification process. Investig. Ophthalmol. Vis. Sci..

[25] Misra D., Booth S.L., Crosier M.D., Ordovas J.M., Felson D.T., Neogi T. (2011). Matrix Gla protein polymorphism, but not concentrations, is associated with radiographic hand osteoarthritis. J. Rheumatol..

[26] Azuma K., Inoue S. (2019). Multiple modes of vitamin K actions in aging-related musculoskeletal disorders. Int. J. Mol. Sci..

[27] Neogi T., Felson D.T., Sarno R., Booth S.L. (2008). Vitamin K in hand osteoarthritis: Results from a randomised clinical trial. Ann. Rheum. Dis..

[28] Chaudhary P., Janmeda P., Docea A.O., Yeskaliyeva B., Abdull Razis A.F., Modu B., Calina D., Sharifi-Rad J. (2023). Oxidative stress, free radicals and antioxidants: Potential crosstalk in the pathophysiology of human diseases. Front. Chem..

[29] Dunlap B., Patterson G.T., Kumar S., Vyavahare S., Mishra S., Isales C., Fulzele S. (2021). Vitamin C supplementation for the treatment of osteoarthritis: Perspectives on the past, present, and future. Ther. Adv. Chronic Dis..

[30] Chin K.Y., Ima-Nirwana S. (2018). The role of vitamin E in preventing and treating osteoarthritis—A review of the current evidence. Front. Pharmacol..

[31] Gupte P.A., Giramkar S.A., Harke S.M., Kulkarni S.K., Deshmukh A.P., Hingorani L.L., Mahajan M.P., Bhalerao S.S. (2019). Evaluation of the efficacy and safety of capsule longvida^®^ optimized curcumin (solid lipid curcumin particles) in knee osteoarthritis: A pilot clinical study. J. Inflamm. Res..

[32] Shep D., Khanwelkar C., Gade P., Karad S. (2019). Safety and efficacy of curcumin versus diclofenac in knee osteoarthritis: A randomized open-label parallel-arm study. Trials.

[33] Li G., Cheng T., Yu X. (2021). The Impact of Trace Elements on Osteoarthritis. Front. Med..

